# Undergraduate Students’ Experiences of a Community-Engaged Learning Course: A Mixed-Methods Study

**DOI:** 10.1177/10538259241309640

**Published:** 2025-01-01

**Authors:** Abby Maxwell, Julia Yates, Kate Pfingstgraef, Tara Mantler

**Affiliations:** 1 School of Health Studies, Faculty of Health Sciences, The University of Western Ontario, London, Ontario, Canada; 2 Health and Rehabilitation Sciences Program, Faculty of Health Sciences, The University of Western Ontario, London, Ontario, Canada

**Keywords:** community-engaged learning, experiential learning, undergraduate students, mixed-methods, elementary education

## Abstract

**Background:**

Undergraduate student engagement increases the quality of education. Community-engaged learning (CEL) courses are one way to promote engagement and involve students collaborating with community partners to achieve a common goal by applying course knowledge to real-world issues.

**Purpose:**

This study evaluated: (a) the relationship between CEL-related student learning outcomes (SLOs) and attitudes toward CEL courses before taking one; (b) CEL-related SLOs among undergraduate students before versus after taking a CEL course; and (c) the lived experiences of students who participated in their first CEL course.

**Methodology:**

Pre- and post-course surveys and focus group data were collected. Survey data were analyzed via correlations and dependent groups *t*-tests, while inductive content analysis was employed to analyze focus group data.

**Findings:**

Findings revealed a significant correlation between students’ opinions toward the benefits of taking a CEL course and their CEL-related SLOs and a statistically significant positive difference between student growth and achievement before compared to after completing a CEL course (*t* = 2.6778, *p* = .0123). Students also expressed the benefits of taking CEL courses, including community impacts, conduciveness to learning preferences, and skill development.

**Implications:**

CEL courses are a means to improve students’ motivation, achievement, and skill acquisition for future career preparedness.

## Introduction

The learning disruptions caused by the COVID-19 pandemic had detrimental effects on the elementary education system, with both elementary students and teachers being adversely impacted (UNESCO, 2023). School systems were faced with the near-constant vacillation between emergency remote learning and in-person instruction, resulting in many barriers to students’ ability to succeed academically, socially, and emotionally (Sansone et al., 2021; Yates et al., 2024). Together, these consequences of the pandemic have left achievement gaps among elementary school students, wherein classrooms now have students spanning multiple grade levels (Sansone et al., 2021). Multi-grade classrooms may be challenging as teachers must modify the learning environment for students of various learning levels, yet are facing an ongoing lack of funding, resources, and available time to modify their materials (Hodgson-Bautista et al., 2022). These ongoing constraints to teachers’ time and resources may also contribute to less availability for one-on-one learning or attending to unique student needs, which may be required in multi-grade classrooms (Chu et al., 2020).

Educational deficits, including limited resources for teachers and minimal individualistic support for students, were not only experienced at the elementary level, but as the COVID-19 lockdown progressed, university professors also had an increasingly challenging time keeping students engaged. Specifically, there was a lack of ‘positive participation’ which is seen as effort, attention, and persistence during the initiation and completion of learning activities (Hendrick et al., 2023). A mixed-methods study of university students in the Netherlands (*n* = 228) showed that ‘positive participation’ suffered during a lack of a shared physical space, as was experienced during the pandemic, and there was growing uncertainty about university policies and the quality of students’ personal development, all of which impacted students’ academic engagement (Hendrick et al., 2023). The measures put in place during the COVID-19 pandemic removed students from traditional classroom settings and placed them in a virtual space, where they completed their studies from home or had to miss class to quarantine when they showed symptoms of COVID-19, which ultimately impacted their academic engagement by decreasing the time spent in optimal learning environments (e.g., classrooms with teacher support) which may have contributed to gaps in learning among students (UNESCO, 2023). This engagement is critical to not only the completion of academic tasks but also the quality of learning and performance of students (Fredricks et al., 2004), which further emphasizes the need for behavioral, emotional, and cognitive engagement through various interventions.

One way that universities have brought engagement and educational opportunities back into both elementary and university classrooms is through using community-engaged learning (CEL). CEL involves a method of instruction where classroom teachings are applied to a real-world scenario, providing university students with an opportunity to gain new perspectives by working alongside local partners to achieve a common goal (Brabazon, 2019). A recent review by Seaman et al. (2019) observed the historical trajectory of CEL courses through eight articles, highlighting the presence of many benefits of CEL courses for university students. These benefits included the development of critical thinking, communication, problem-solving, and team-building skills, ultimately allowing students to introduce theoretical perspectives into their learning (Seaman et al., 2019). In the case where undergraduates are partnered with elementary children, past research has also seen benefits. Specifically, in a study by Meyers (2009) wherein undergraduate students worked directly with at-risk children in the community through a service-learning course, students reported personal growth and increased civic engagement. When students are involved in real world partnerships where they can put course material into practice, they perform better academically, have higher graduation rates, and are more satisfied with their post-secondary experience (Meyers, 2009). CEL courses and projects can also affect students on social, moral and emotional levels where, for example, they may develop greater cultural and racial understanding (Meyers, 2009).

The concept of learning by experience has long been used in classrooms of all age levels as a method to engage students in reflective and thoughtful processes beyond traditional lectures (Walker & Rocconi, 2021). In most circumstances, this experiential format of learning can have extremely beneficial impacts on students’ reflection and conceptualization of classroom content (Mcleod, 2024). As such, CEL should be employed to promote the learning and comprehension of course curricula among undergraduate students. Despite this, there is currently a gap in the literature assessing how post-secondary students feel about CEL courses before having taken one in terms of engagement, enjoyment, and understanding. By studying the impacts of CEL courses on the experiences, enthusiasm, and attitudes of university students before and after taking one, we can better assess how to implement and expand the usage of CEL courses. With the knowledge of these promising impacts from previous research on CEL (e.g., the development of critical thinking, communication, problem-solving, and team-building skills; Seaman et al., 2019), it is important to assess not only the ways in which CEL courses can impact students’ learning, but also their enjoyment and enthusiasm toward the course and their impacts on the broader community, as students are more likely to engage with and benefit from the curriculum while also contributing to a positive classroom environment. The unique nature of this study allowed the exploration of students’ opinions and attitudes toward CEL courses before and after taking a CEL course for the first time, which to the authors’ knowledge is the first study of its kind performed in Canada. Observing the differences in student opinions toward CEL courses over time could be extremely beneficial in informing educational institutions about CEL courses and their possible benefits for students and the broader community alike. To this end, the purpose of this mixed-methods study was to evaluate: (a) the relationship between CEL-related student learning outcomes (SLOs; i.e., lifelong learning, application of knowledge and skills, collaboration, and structured reflection) and attitudes toward CEL courses before taking one; (b) CEL-related SLOs among undergraduate students before versus after taking a CEL course; and (c) the lived experiences of students who participated in their first CEL course.

## Methods

This mixed methods design consisted of pre-course data collected via surveys in September 2023, and post-course data collected via both surveys and focus groups during January 2024. Ethics approval for this study was received from the host institution (NMREB #122740).

### Design

In September 2023, The School of Health Studies (SHS) LEARN Lab was launched, forging a partnership between approximately 400 students in a second-year university course and elementary teachers across Ontario. The goal was to create an open-access repository of short, evidence-based, developmentally appropriate, and engaging health resources to bridge the learning gap in elementary school classrooms while simultaneously improving the academic experience of undergraduate students. As part of this innovation, undergraduate students were taught the foundations of biological, cognitive, and social development among children from the prenatal period to adolescence. The CEL project, which was a required course component, then paired students with elementary school teachers to create customized health-related resources for use in the classroom. The intended benefits of this project were two-fold: (a) undergraduate students had the chance to experience the benefits of bringing their classroom knowledge into the real world to prepare requested materials; and (b) elementary teachers received customized health-related resources to fit the specific needs of their elementary classroom.

### Participants

To be eligible to participate in this study, students had to be enrolled in a *Health Issues in Childhood and Adolescence* (i.e., *HS2700*) course at a large university in Southwestern Ontario in the Fall 2023 semester and understand the English language.

### Procedures

Participants were recruited via in-class announcements made by a research team member external to the course. Upon scanning the QR code located on the recruitment graphics, interested participants were re-directed to qualtrics^XM^ (Qualtrics, Provo, UT), an online survey platform, to provide voluntary, informed consent and complete the baseline survey. Students who completed this survey had the option to enter a draw for one of three $50 Amazon e-gift cards. Participants who consented to being contacted for a follow-up during the baseline survey were sent the follow-up survey link via email in January 2024. Through completing this survey, participants could express interest in a focus group.

### Materials

#### Surveys

The online pre-course survey included (a) demographic questions; (b) global questions related to students’ attitudes toward CEL courses; and (c) the previously validated pre-course version of the Experiential Learning Study Survey (Walker & Rocconi, 2021). The online post-course survey included global questions related to post-course attitudes toward CEL courses and the post-course version of the Experiential Leaning Survey (Walker & Rocconi, 2021).

##### Demographics

A series of closed-ended questions pertaining to the students’ age, sex, gender, ethnicity, enrollment status, and program and year of study were asked.

##### Global Questions

A series of global questions were asked to gauge students’ enthusiasm toward taking a CEL course for the first time. A similar series of questions were asked in the follow-up survey, which assessed students’ enthusiasm and attitudes toward CEL courses after completing one for the first time. See Table 1 for an overview of global questions and response options from pre- and post-course surveys.

**Table 1.** Global Questions and Response Options From Pre- and Post-Course Surveys.

Global question	Response options
Pre-course survey	
1. I am looking forward to the CEL component of this course.	Strongly Agree, Agree, Neither Agree nor Disagree, Disagree, Strongly Disagree
2. I think there are benefits to taking a CEL course.	Strongly Agree, Agree, Neither Agree nor Disagree, Disagree, Strongly Disagree
3. I am concerned about the CEL component of this course.	Strongly Agree, Agree, Neither Agree nor Disagree, Disagree, Strongly Disagree
4. Please rate your level of enthusiasm for taking *HS2700* on a scale of 0–10 (0 denoting not enthusiastic at all; 10 denoting very enthusiastic).	0–10 rating scale
5. Please rate your level of enthusiasm for the CEL component of *HS2700* on a scale of 0–10 (0 denoting not enthusiastic at all; 10 denoting very enthusiastic).	0–10 rating scale
Post-course survey	
6. I am glad to have been part of the CEL component of this course.	Strongly Agree, Agree, Neither Agree nor Disagree, Disagree, Strongly Disagree
7. I think there are benefits to taking a CEL course.	Strongly Agree, Agree, Neither Agree nor Disagree, Disagree, Strongly Disagree
8. I have a more positive attitude toward CEL courses after taking *HS2700*.	Strongly Agree, Agree, Neither Agree nor Disagree, Disagree, Strongly Disagree
9. My concerns about the CEL component of this course were validated through my experience taking the course.	Strongly Agree, Agree, Neither Agree nor Disagree, Disagree, Strongly Disagree
10. My final grade was within the range I expected for this course.	Strongly Agree, Agree, Neither Agree nor Disagree, Disagree, Strongly Disagree
11. Please rate your level of enthusiasm for future CEL courses on a scale of 0–10 (0 denoting not enthusiastic at all; 10 denoting very enthusiastic).	0–10 rating scale

##### Experiential Learning Student Survey

The 16-item Experiential Learning Survey is a previously validated scale, consisting of four subscales related to student growth and achievement (Walker & Rocconi, 2021). Using pre- and post-course experience surveys relating to Kolb's experiential learning cycle (Kolb, 1984), this instrument measures undergraduate students’ perceptions of learning before and after completing some form of CEL. Students were asked to rate their agreement to the statements on a 7-point Likert scale, with options ranging from strongly disagree to strongly agree. Strong invariance of the instruments over time has been recorded and used to measure CEL-related SLOs, separated into four subscales: (a) lifelong learning (i.e., Experiential Learning Student Survey questions 1–4); (b) application of knowledge and skills (i.e., Experiential Learning Student Survey questions 5–10); (c) collaboration (i.e., Experiential Learning Student Survey questions 11–13); and (d) structured reflection (i.e., Experiential Learning Student Survey questions 14–16). The internal consistency of these instruments was previously measured using McDonald's (1999) *ωt* coefficient which offers a more precise depiction of variability during the point estimation process compared to Cronbach's alpha (Dunn et al., 2013). All SLO's other than the “collaboration” factor, which displayed moderate internal consistency, have strong internal consistency above *ωt* = .700 (Walker & Rocconi, 2021).

#### Focus Groups

The purpose of the focus groups was to capture the lived experiences of students who participated in their first CEL course. Focus groups took place via Zoom with a moderator and notetakers lasting approximately 30 minutes in length each. The focus groups followed a topical interview guide with questions surrounding the lived experiences of taking a CEL course (e.g., impacts of CEL course on engagement with material and academic achievement). All students who consented to participating in a follow-up focus group were invited to do so (i.e., no special selection of students based on pre-determined factors were made by the research team). Please see Appendix A for the Focus Group Guide. The focus groups were audio-recorded and transcribed verbatim by Zoom (Zoom, 2024) and checked for accuracy and anonymized by a member of the research team. To enhance readability, the research team removed instances of “um,” “like,” and word repetitions (Corden & Sainsbury, 2006).

### Analytic Strategy

#### Surveys

All data analyses were completed using R (Version 4.2.2; The R Foundation for Statistical Computing Platform, 2022). Participants were included in analyses if they completed a minimum of 18 of the 25 total questions in the post-course survey (i.e., responses with <30% answered questions were removed from the study; Dong & Peng, 2013). No participants were removed from analysis due to missingness. Measures of central tendency and dispersion were computed for all demographic, global, and scale questions.

**Experiential Learning Student Survey.** Scale questions were scored on a scale from 1 (strongly disagree) to 7 (strongly agree). An average of the 16-items was calculated, reflecting students’ CEL-related SLO scores (Walker & Rocconi, 2021). For each of the four subscales (i.e., lifelong learning, application of knowledge and skills, collaboration, and structured reflection), average sub-scale scores were also calculated. Higher scores indicate a positive appreciation for CEL courses as well as an increased ability to meet course learning outcomes.

**Relationship Between SLOs and Attitudes Toward Course.** Pearson Product moment correlations were conducted to assess the relationship between average scores on CEL-related SLOs and students’ attitudes and enthusiasm toward the course pre-course delivery. Five separate correlations were computed between students’ average scores on the CEL-related SLOs and: (a) outlooks toward the CEL component of the course; (b) enthusiasm toward taking *HS2700*; (c) enthusiasm toward the CEL component of *HS2700*; (d) opinions toward the benefits of taking a CEL course; and (e) concerns toward the CEL component of the course.

**Differences Between Pre- and Post-Course Delivery**. The differences in student growth and achievement from CEL courses after having completed a CEL course for the first time were investigated using dependent groups *t*-tests. The dataset met the assumptions of a dependent groups *t*-test. To adjust for multiple comparison bias between the four SLO subscales, a Bonferroni correction was employed (*α*/4 = 0.0125).

#### Focus Groups

Quirkos qualitative analysis software (Quirkos, 2021) was used to organize the focus group transcripts. An inductive content analysis approach (Patton, 2015) was used following the method for thematic analysis as outlined by Braun and Clarke (2006). Four researchers independently and simultaneously familiarized themselves with the data by reading and re-reading the transcripts while making notes (Braun & Clarke, 2006). Multiple researchers were used to support confirmability (Guba & Lincoln, 1989). Next, the researchers generated the initial codes by coding the entire dataset and then collating the codes into potential themes (Braun & Clarke, 2006). The four researchers were split into dyads and were assigned one transcript in which each researcher independently created a preliminary codebook based on the transcript. The dyads then met to discuss their findings. Following, both dyads came together in a larger meeting to synthesize the two preliminary codebooks into one final codebook. Through this process they ensured the themes related to the coded extracts (step 1) and then to the entire dataset (step 2), and refinements were made to the themes and definitions as necessary (Braun & Clarke, 2006). This codebook was then used to complete analysis, with each transcript being analyzed by two researchers. Subsequently, Quirkos files were merged and exported.

## Results

### Demographics

In total, 196 pre-course survey responses were collected, with 57 responses being removed due to lack of completeness. Pre-course data was therefore included for 139 students (*M*_age_ = 19.09 years, *SD* = 0.72). The majority of participants identified as female (*n* = 117, 84.16%), in their second year of undergraduate studies (*n* = 108, 77.70%). Most participants reported being enrolled in *HS2700* as a mandatory component of their degree (*n* = 135, 97.12%) in the Health Studies program (*n* = 133, 95.68%), with all being enrolled full-time (*n* = 139, 100.00%). Full pre-course demographic information can be found in Table 2.

**Table 2.** Sociodemographic Characteristics of Participants.

Participant characteristics	Pre-course survey		Post-course survey		Focus groups	
	*n*	%	*n*	%	*N*	%
Age, *M (SD)*	19.09 (0.72)		19.20 (0.71)		19.60 (0.80)	
Sex						
Female	117	84.16	26	86.67	6	85.71
Male	21	15.12	4	13.33	1	14.29
Prefer not to say	1	0.72	-	-	-	-
Gender identity						
Woman	115	82.73	26	86.67	6	85.71
Man	21	15.12	4	13.33	1	14.29
Prefer not to say	3	2.15	-	-	-	-
Ethnicity						
African Origins	2	1.44	-	-	-	-
Asian Origins	48	34.50	8	26.67	2	28.57
Caribbean	1	0.72	-	-	-	-
European	52	37.41	18	60.00	3	42.86
Latin, Central, and South American Origins	2	1.44	-	-	-	-
Multi-racial or mixed racial identity	17	12.23	2	6.67	2	28.57
Other North American Origins	10	7.19	2	6.67	-	-
Self-Identify	6	4.32	-	-	-	-
Prefer not to say	1	0.72	-	-	-	-
Year of study						
Year 2	108	77.70	20	66.67	4	57.14
Year 3	26	18.71	9	30.00	2	28.57
Year 4	4	2.88	1	3.33	1	14.29
Year 5+	1	0.72	-	-	-	-
Mandatory enrollment in course						
Yes	135	97.12	30	100.00	7	100.00
No	4	2.88	-	-	-	-
Program						
Health studies	133	95.68	29	96.67	6	85.71
Other	7	5.04	1	3.33	1	14.29
Enrollment status						
Full-time	139	100.00	30	100.00	7	100.00

*Note.* Age was measured in years. Sex refers to the participant's biological sex at birth.

The post-course survey was completed by 30 participants (*M*_age_ = 19.2 years, *SD* = 0.71). The majority of participants were in their second year of undergraduate studies (*n* = 20, 66.67%), identified as female (*n* = 26, 86.67%), and were enrolled in the Health Studies program (*n* = 29. 96.67%). All students in this study were full-time students enrolled in *HS2700* as a mandatory component of their degree. Full post-course demographic information can be found in Table 2.

A total of four focus groups occurred, ranging in size from 1 to 2 participants. The focus groups consisted of seven participants (*M*_age_ = 19.6 years, *SD* = 0.80). The majority of the participants identified as female (*n* = 6; 85.71%) and were in their second year of undergraduate studies (*n* = 4; 57.14%). All participants reported being enrolled in *HS2700* as a mandatory component of their degree and were enrolled full-time, and most of the participants were in the Health Studies program (*n* = 6; 85.71%). Complete demographic information of the focus groups can be found in Table 2.

### Global Questions

Responses to global questions revealed that pre- and post-course, students had positive attitudes toward and were enthusiastic about CEL courses. Among the questions asked in the pre-course survey, the highest scoring questions were “I am looking forward to the CEL component of this course” and “I think there are benefits to taking a CEL course.” Among the global questions asked in the post-course survey, the highest scoring questions were “I am glad to have been part of the CEL component of this course” and “I think there are benefits to taking a CEL course.” The average scores for each global question can be found in Table 3.

**Table 3.** Descriptive Statistics of Global Questions.

Global question	*M*	*SD*
Pre-course survey		
I am looking forward to the CEL component of this course.	3.84	0.87
My level of enthusiasm for taking *HS2700* is:	7.34	1.54
My level of enthusiasm for the CEL component of *HS2700* is:	6.46	1.90
I think there are benefits to taking a CEL course.	4.09	0.65
I am concerned about the CEL component of this course.	3.24	0.86
Post-course survey		
I am glad to have been part of the CEL component of this course.	4.30	0.99
My level of enthusiasm for future CEL courses is:	7.90	2.21
I think there are benefits to taking a CEL course.	4.27	0.94
I have a more positive attitude toward CEL courses after taking *HS2700*.	4.10	1.00
My concerns about the CEL component of this course were validated through my experience taking the course.	3.57	0.86
My final grade was within the range I expected for this course.	3.70	0.84

### Relationship Between SLOs and Attitudes Toward Course

There was a significant correlation between students’ opinions toward possible benefits of taking a CEL course and their total SLO scores. The strongest correlation was found between enthusiasm toward the course (*r* (137) = 0.41, *p* = .00000047) and outlooks toward the CEL component of the course (*r* (137) = 0.40, *p* = .0000013). See Table 4 for full correlational analyses.

**Table 4.** Relationship Between SLOs and Attitudes Toward the Course.

Variable	*n*	*r*	*R^2^*	*df*	*p*
1. Outlooks toward the CEL component of the course.	139	0.40	0.16	137	.0000013**
2. Opinions toward the benefits of taking a CEL course.	139	0.37	0.14	137	.0000089**
3. Concerns toward the CEL component of the course.	139	0.19	0.037	137	.024*
4. Enthusiasm toward taking *HS2700*.	139	0.41	0.17	137	.00000047**
5. Enthusiasm toward the CEL component of *HS2700.*	139	0.39	0.15	137	.0000020**

*Note.* Question numbers correspond to global questions asked in the baseline survey.

* *p* *<* .05.

** *p* < .001.

### Differences Between Pre- and Post-Course Delivery

The dependent groups *t*-tests revealed a positive significant difference between students’ average SLO score pre- and post-course. The greatest differences were reported in SLO subscale 2: solving real-world problems, and SLO subscale 3: collaboration (see all statistical results in Table 5).

**Table 5.** The Difference in Pre- and Post-Course SLO Scores.

Item	*M* (*SD*)	*M*_D_	*t*(30)	*p*	Lower 95% CI	Upper 85% CI
SLO Subscale 1: Lifelong Learning	6.29 (0.62)	0.29	1.730	.0945	−0.637	0.0534
SLO Subscale 2: Solving Real-World Problems	5.79 (0.63)	0.51	2.547	.0167	−0.913	−0.0989
SLO Subscale 3: Collaboration	6.48 (0.40)	0.28	2.342	.0263	−0.520	−0.0352
SLO Subscale 4: Structured Reflection	5.93 (0.84)	0.05	0.310	.759	−0.338	0.249
Average SLO Score	6.08 (0.45)	0.32	2.678	.0123*	−0.568	−0.0756

*Note. M* = mean; *SD* = standard deviation; *M*_D_ = mean difference; CI = confidence interval.

* *p* ≤ .0125.

### Thematic Results

Four themes related to undergraduate's experiences of CEL courses emerged from the focus group data: (a) community impact fostered motivation; (b) conduciveness to learning preferences; (c) relevance to course concepts; and (d) future impacts. See Table 6 for an overview of the themes and associated definitions.

**Table 6.** Themes and Associated Definitions.

Theme	Definition
(1) Community impact fostered motivation	The CEL project allowed undergraduates to aid real world problems in their community which fueled inspiration and motivation back to the actual course.
(2) Conduciveness to learning preferences	The application of course concepts and alternative course assessments gave students the opportunity to learn through their preferred method.
(3) Relevance to course concepts	The CEL project related directly to the course objectives and content.
(4) Future impacts	The impacts of the CEL project on future decisions, recommendations, and skills.

#### Community Impact Fostered Motivation

The CEL project impacted the community, and this realization supported students learning and served as a source of motivation for them. As their projects began, students quickly realized “…what [they] were doing [could] be applied to real life because that was the whole point of it, right?” (N3). Students felt that the CEL component allowed them to “do this really cool project and actually apply our work to somewhere outside of ourselves” (N1). Focusing on the idea of *outside of ourselves*, students explained how community impact was important to them as a means to contribute to issues in the real world and a way to provide tangible support to the community beyond the classroom. For example, one student shared “I think just everyone knows how difficult it is to be a teacher these days and I think that it was cool that we were making relevant resources for students to help them out” (N7). This student felt the creation of their resource was a small way to help support teachers in succeeding following the challenging circumstances of the COVID-19 pandemic, and this ability to provide support motivated them.

Other participants reflected similarly in terms of the source of motivation behind creating the resource, sharing that the most impactful part of participating in a CEL course was “having other people use your resource and knowing that somebody out there actually benefited from something that we made” (N2). Overall, students felt that creating resources that could be used to help other people was an enriching part of the CEL experience and many of the participants saw the CEL component as an addition that was “really helpful and kind of an incentive to wanna do the course” (N3). The CEL component inspired students to attend class regularly and be more invested in the content being taught to then impact the community. A student elaborated on this saying:

I think it engage[d me] more and [made me] want to go to class and participate in discussions and tutorial because then it kind of helped me know and make sure that I knew what I was doing when I was doing the project, and not just kinda shooting at a whim, I wanted to go ‘cause I wanted to do well. (N1)

Not only did the project make the students want to attend class but they also appreciated how the CEL application of *HS2700* made the course unique and more enjoyable then other courses: “for example, some of my online classes, I didn't really have that engaging component so I wouldn't spend as much time learning the material as I would have if there was an engaging component” (N2). Other students agreed that the engaging component was truly unique to this course and stated, “I feel like we don’t really get an opportunity to do that in a lot of classes” (N1). Overall, the CEL project had positive impacts on students regarding the ability to interact with the community which fostered their sense of motivation toward the course and left them thinking, “I wanna do well and I want to show that I've learned something in this course” (N1).

#### Conduciveness to Learning Preference

Participation in the CEL project was conducive to student learning as the application of course concepts and alternative course assessments made learning fun. Many students commented on the benefits of being able to apply the course concept tangibly. For example, one student stated, “I think it definitely increased my understanding ‘cause it allowed like me to… put the things I learned into an application sense” (N6). Another student emphasized that being able to apply what they knew, as opposed to simply memorizing the content, motivated them in “wanting to learn and wanting to do well, so then [they] ended up doing well, because [they] put in the effort to do well” (N1).

The CEL project was further conducive to learning for some students as it allowed them to absorb and interact with the content in a way that worked better for them. One participant described how having alternative course assessments benefited them in comparison to the typical testing style of second-year courses that primarily includes quizzes and exams:

I think that it really helped my mark in this course because there was something tested on other than exams, and I find that I perform more better on projects or on assignments that go through a long period of time that I can actually learn the course material more better than it being a cram kind of thing, and having to show my, what I know through a two hour assessment. (N2)

Cramming is not the only issue students may face when their knowledge is being tested through exams but many also found they “don’t always do well in midterms and exams, so to have an assignment it gave me that security” (N3). Another student agreed with this statement:

This assignment could alleviate some stress from my midterms and finals like [other participant in focus group], I don't tend to do very well on the midterms or finals, but I like doing assignments because they are almost a guaranteed mark to help boost your grade. (N4)

For some students, the project was conducive to their learning preference as completing the project did not feel like learning at all: “[the CEL project] was a de-stressor in some ways because it was a fun project that we got to do for the class, it wasn't just quizzes or tests or something” (N7). When classes only evaluate students understanding with tests or exams it may cause what one student referred to as test anxiety and how “that just diminishes my capabilities” (N6). A participant reflected on their experience of the *HS2700* course and demonstrated how this statement may be true, “so I feel like knowing that I already had a pretty good mark going into the exam, it made me only want to do better on the exam and I feel like I performed well on that as well” (N5).

#### Relevance to Course Concepts

Participants had conflicting experiences related to the relevance of their CEL projects to course concepts, with some students reporting that the CEL project related directly to the content which then helped them stay engaged, learn the content better and stay up to date in the course. Other students described the CEL project as not relevant to the course content and therefore requiring additional research.

The CEL project made content relevant, as once concepts were taught and students had the opportunity to apply them, students reported feeling more confident in knowing the material. For example, one participant explained how their group felt after they made their resource:

I think that it really made the material more real for us or more applied like we could apply it better to our things that we were doing in our project. Um yeah, I think it really helped with the learning aspect and having real examples and stuff. (N7)

Beyond helping to learn content, the CEL component also helped to solidify the learning. For example, when participants knew the content would be used in the CEL project, it helped facilitate retention of the information. For some students it “kind of created this cycle of productivity and I found that I was able to get a lot more done and retain a lot more because of the CEL project” (N4).

For some students, the connection to course concepts was not as explicit, compared to other groups’ projects. As such, some students felt that their topic required more external research:

I wish that the topics for the CEL projects related a little bit more to the course content. I feel like both of the ones that I had, I had global citizenship, and we didn’t really ever talk about global citizenship in class. So it's definitely a little bit more difficult to incorporate that stuff into our presentation and also, all the research that I had to do for that assignment was new. Like it wasn’t from the lecture slides. So it felt like I wasn’t reviewing things I had already learned. Like this was something completely different that I was doing for this class. (N5)

For others, external research was the starting place for them to find relevancy with course concepts, a process which allowed them the opportunity to draw connections between course material. One participant explained that their group “had to do external research for almost everything relating to global citizenship to make up our song … And then after… we went back into the text and tried to find things that could relate” (N6).

#### Future Impacts

The participants’ futures were impacted by the CEL project in terms of increasing the likelihood that they would take another CEL course and through the development of essential skills to use in their future. Additionally, all participants agreed that in the future, they would recommend a CEL course to others after their experience in *HS2700*.

For many participants, the CEL project had such a positive impact on their experience of the course that they state, “I would definitely take another CEL course” (N6). One participant even made the claim that it was such a great experience that they think it should be mandatory, “and [they would] love to take the opportunity to do another one or something similar because [they] see the importance of something like this and, yeah, it was just a really overall good time” (N1). Some participants had initial worries about the CEL component but after the project was completed their worries were depleted. For example, one student stated, “at first, I was again a little bit worried about it because there was no prior years but once I got into it, I loved it” (N3). The same student went on to state, “I almost switched into environmental CEL promotion, which is a CEL course in Health Sci” (N3). Another student had very similar initial concerns surrounding the fact that the CEL integration was new to the course, however their opinion changed upon personal completion of the CEL project:

I think for a first year I think it was great. I was able to learn a lot from it and I definitely do want to take more courses that have CEL projects because I like being able to apply course content in a community engaged way that would actually see change and I think if there's the opportunity, I would definitely like to take another CEL project course. (N4)

A different participant displayed a less worrisome initial view of the CEL component compared to the above examples, yet still had a similar opinion after completion, “I don't think I really knew too much about CEL projects before I went in, so I feel like it was a good introduction to them and I'm definitely looking into more for my later years” (N7). It was demonstrated that no matter their apprehensions about a CEL component before the actual completion of the CEL project, participants ended up enjoying it and wanting to take more courses with a CEL component.

Despite having completed the course, students explained how they would carry lessons learned from this experience with them into the future. One student described their experience of this, demonstrating how it's a lot more “independent in a sense where you don't have the teacher telling you study this, and you’re tested on that. So it definitely impacted me and prepared me for a future in health science and public health” (N6). Another student explained that “…you never know what can come out of it, and maybe you know you kind of have some professional realizations or you learn something new, or have a new reflection of yourself in your life” (N2). Across the board, students shared examples of “professional realizations,” with one student saying, “I feel like I could like translate [skills learned from the CEL component] into a future job or something like that” (N3). Students also shared that the CEL course provided them with valuable experiences to talk about in future interviews, explaining how talking about the impacts of a CEL component of a course “is way more impactful then just saying you wrote an exam for this course” (N5). Another student agreed that the CEL course had such an impactful influence on their academic career that they would include it as part of their grad school applications. They explained it as something they could “use for [their] future and make future endeavors more likely to happen” (N6).

Not only did the participants gain skills for their futures and want to take more CEL courses themselves, all the students who participated in the focus groups agreed that they would recommend taking a CEL course to others, therefore possibly impacting other students’ futures. One student shared that “I would recommend it to anyone ’cause I think it was a great part of the course… I feel like it was a really good learning experience and really good practical application of health sciences” (N6). Another participant agreed and also touched on the idea of recommending because of the application component:

I would definitely recommend this course to other people as well. Just ‘cause, whenever my friends hear me talking about like 2700 ’cause I was able to apply it so much to everything that I see, they really were interested so I think a lot of people would be interested in a CEL course style. (N2)

One student emphasized the positive component of conduciveness to learning that a CEL component brings, explaining how “you’re applying things to the real world and you get that assignment component if you're not necessarily the best with exams and testables” (N3), being the reason they would recommend a CEL course to others.

## Discussion

Students reporting higher levels of enthusiasm before the course had higher CEL-related SLO scores, as evidenced through the correlational analyses. Additionally, students had an overall increase in CEL-related SLO scoring from pre- to post-course. These findings are reflective of stronger skills related to critical thinking, ethical reasoning, inquiry and analysis, global learning, and integrative learning, likely resulting in an increased ability to meet the course's learning outcomes and growth in students’ learning and enjoyment (Walker & Rocconi, 2021). These findings were reflected in focus group themes wherein students expressed that community involvement fostered their motivation, the CEL project made the course conducive to their individual learning preferences, and was relevant to course concepts, which combined, contributed to their academic success now, while also helping them develop skills that could be useful in future careers.

The correlations revealed moderately strong correlations between students’ opinions toward possible CEL course benefits and their CEL-related SLOs, indicating a broader understanding of their own learning and impacts on their community. This could be understood using the concept of metacognition, which is an awareness and control for one's own thinking and learning (Stanton et al., 2021). As mentioned in a study by Pomerantz (2006), when a learner is actively engaged in the learning process, they are more likely to meet the learning outcomes. This efficiency in learning could largely be explained by metacognition, when students are able to identify concepts, they do not understand and select appropriate strategies for learning those concepts (Stanton et al., 2021). Prior to engaging in the CEL components of the course, students enrolled in this course all may have had varying levels of metacognition, thus influencing their ratings of the possible benefits that a CEL course may have on their learning. Another study suggests that metacognition should be implemented into group work settings, allowing students to observe their peer's hypothesis, predictions, explanations, and interpretations of problems (Choi & Kuo, 2009). This social form of metacognition can stimulate metacognition in one another, leading to improved outcomes for all students involved. Thus, it can be said that regardless of prior levels of metacognition, helping students develop their metacognition by providing opportunities and activities that support its expansion, students can learn more and perform better in courses (Stanton et al., 2021). This underscores the importance of integrating group work and CEL into the undergraduate curriculum as it not only fosters academic achievement among students but may also instill a deeper sense of metacognition and holistic skill development—two attributes that may better position students when entering the workforce post-graduation.

Students reported increases from pre- to post-course on all four SLO subscales (i.e., lifelong learning, solving real-world problems, collaboration, and structured reflection). This indicates a positive appreciation for CEL courses and student recognition of their learning, personal development, growth, and contributions to the broader community. The subscale scores of the Walker and Rocconi (2021) survey, designed specifically to measure undergraduate students’ perception of their learning in an experiential learning context, show a positive increase in scores upon a strong reception of the integration of CEL. In the broader literature, it has been recognized that CEL courses allow students to engage more meaningfully with their course content, highlighting the importance for the integration of CEL into courses, which could further allow students to engage with and enjoy their academic pursuits.

As evidenced through students’ increased scores related to solving real-world problems from pre- to post-course and anecdotes from the focus groups, coursework having community impacts fostered feelings of motivation among students. Specifically, this sense of contributing to the community inspired and motivated undergraduate students to complete the necessary work, contributed to their academic success, and fostered the development of skills that may prove to be useful for their future career aspirations (e.g., collaboration, communication). These findings are well-established in past research. Cefalu and Kney (2020) reported that when students contributed to real-world issues and felt that they were making an impact, the results were intrinsically rewarding and provided a sense of inspiration. Kostell et al. (2021) furthered this work by reporting that collaborative and transdisciplinary approaches, such as CEL courses, can promote community engagement among undergraduate students. More specific to the CEL project conducted in this course, a scoping review on the impacts of undergraduate student-developed resources on elementary students and teachers concluded that undergraduate-student created resources positively impacted the elementary school system (Pfingstgraef et al., 2024). Combined, this research demonstrates how the contributions of undergraduates to the elementary school system can be beneficial for both themselves, in terms of professional development, as well as the elementary students and teachers receiving the custom resources.

The present study confirmed the importance of collaboration as one of the most critical components of CEL, allowing students to work together to formulate ideas and actionable solutions to the real-world problem at hand. Post-course completion, students reported the highest scores on the collaboration subscale, which measures students’ abilities to collaborate with other students in a group. A study by Hou (2014) of 162 students at the University of Georgia was conducted to assess students’ abilities to learn and work collaboratively in groups in a CEL course. Similarly to the present study, Hou (2014) reported positive student evaluations on the CEL course, with students commenting specifically that collaboration facilitated their critical thinking skills, increased their self-confidence and self-awareness, and allowed for a shared appreciation for the real-world relevance of the course. Combined, these results indicate the importance of CEL for expanding students’ academic skills and abilities to work collaboratively. By prioritizing these aspects via CEL courses, educational institutions can encourage students to be more engaged and socially collaborative, setting them up for success in their future careers and community-related contributions. Further, following the extensive online learning that occurred during the COVID-19 pandemic, universities may wish to integrate CEL into more courses to increase social connectivity and engagement among students, two aspects that were largely missing during online learning.

CEL is a form of active learning that, based on the results from this study, made *HS2700* more engaging for students and made them appreciative of this unique course component. A study by Surapaneni (2023) of 103 first year medical students integrated an active learning component and reported that this made learning more enjoyable and motivating for students and supported that being able to apply course concepts through alternative course assessments beyond simply administering traditional exams enhances learning. Applying content in unique ways also allowed students to absorb and interact with the content affording the opportunity for deeper learning as concepts came alive, findings that were echoed by focus group participants. Further, a study by Chetty et al. (2019) found that a mismatch between teaching and learning preferences can lead to underperformance and student disappointment, highlighting the importance of integrating different learning preferences into a course so that students can learn in a way that best supports their knowledge acquisition. This research supports the integration of CEL projects into courses, wherein students can succeed in different ways that are conducive to their unique abilities.

The relevance of CEL projects to course concepts was another critical aspect of the project to students, as reflected by focus group responses. Evidence related to the importance of CEL project-relevance to course concepts was found in a study by Flaisher-Grinberg (2023), which looked at undergraduate students (*n* *=* 21) in a CEL-based psychology course. Students reported that class content was relevant to their CEL project and therefore increased their comprehension. This reaffirms the present study's findings that when CEL projects are relevant and integrated into pre-existing course curriculum, students are more likely to meet course learning outcomes and be engaged with the material. To ensure effective community-engaged partnerships, universities need to ensure they are providing structures for students to share their past CEL experiences with other students, have mutually beneficial conversations with their community partners, negotiate with their educational/university partners, and receive mentoring and support from both their university and community partners (Cutler & Dorow, 2012). This is an important point to note in terms of implementation and creation of further CEL courses to ensure they succeed in supporting students with knowledge acquisition.

When students complete a CEL course, they learn valuable lessons in which they can carry with them into the future. Students in the present study found the CEL project so valuable that they would take another one and/or recommend it to a peer for this sole reason; however, there is no specific literature that has explicitly stated students who have taken a CEL course in the past would be more likely to take another one or recommend it to others. Although, two studies (Cooper & Kotys-Schwartz, 2013; Shinneman et al., 2020) have indicated that students are more interested in CEL courses than non-CEL courses. Future research should explore the longitudinal course patterns of students after taking a CEL course to investigate if these students did in fact participate in more CEL-based courses and/or if the positive impacts found here extend to other courses and future years of undergraduate education.

### Limitations

The limitations of this study must be considered. Firstly, under half of the class completed the baseline survey and individuals who chose to participate may have differed systematically from individuals who chose not to participate, so the findings might not be able to be generalized to the entire class. Second, social desirability bias must be considered as although the identity and participation of students in the survey and research were kept anonymous, the students were aware that the teaching team would be involved in the research after final course grades were submitted, and as such may have felt some obligations to participate as a result. Third, the post-course survey was only completed by 30 participants and the focus groups by seven, thus limiting the generalizability and representativeness of results; however, the participant pool for this study was finite since participation was only open to students enrolled in *HS2700* in the Fall semester of 2023. Furthermore, since post-course data collection occurred in January 2024 at the start of the new semester, some students may have shifted their focus away from Fall courses. In addition, the relatively low level of demographic variability must be considered, as most students who participated in the post-course survey were female and all students were taking *HS2700* as a required course for their undergraduate degree. Future research should utilize methods such as sampling for longer or targeting diverse groups to ensure results are representative.

## Conclusion

This study explored the attitudes of undergraduate students toward CEL courses before versus after taking a CEL course for the first time and their lived experiences of taking part in their first CEL course. The CEL project fostered motivation through community impact, made the course conducive to multiple learning preferences, and was relevant to course concepts, which supported academic success. These findings highlight the importance for higher education institutions to create and incorporate more CEL opportunities into courses, particularly during earlier years of an undergraduate degree (i.e., first or second year). Future studies should explore the long-term impacts of CEL-based courses on students’ enjoyment of learning, academic success, and course selection patterns.

## Appendix A

### Focus Group Guide

1. To begin today, I’m curious regarding your general impressions toward the CEL component of HS2700, now that you have completed the course?
2. Moving more specifically to talking about engagement with course materials, what was the impact of the CEL course on your overall engagement with the HS2700 course material?
  - Did the CEL component feel relevant to the HS2700 learning outcomes?
  - Did the CEL component increase your understanding of the course content?
3. Transitioning to talk about your achievement within the course, I’m curious of the impact of the CEL component of HS2700 on your academic achievement?
  - Were you happy with your mark in HS2700?
  - Did you feel like the CEL component helped or hindered your mark in HS2700?
  - Did the CEL component cause additional stress related to this course?
4. If we think about the overall purpose of CEL courses, they are meant to provide students with the opportunity to apply their course-based knowledge within real-world settings. In your opinion, in what ways did HS2700 achieve/not achieve this purpose of providing you with the opportunity to apply your course-based knowledge within real-world settings?
  - Impacts on lifelong learning? In what settings?
  - Impacts on solving real-world problems?
  - Impacts on collaboration skills?
  - Impacts on structured reflection (i.e., reflecting on own learning experiences)?
  - Applicability of CEL component to real-world experiences?
5. As a follow up to the last question, what was the impact of having a CEL component in a second year required HS course?
  - Likely to take another CEL course?
  - Likelihood of recommending a CEL course to others?
6. Prior to the CEL course, the overwhelming response of what students were most concerned about was the group work/collaboration aspect of this project. Looking back, what was the impact of the group work component of the CEL course on your overall engagement and/or success with this project?
  - Was there another concern/challenge that ended up being more impactful than the group work?
7. What was the most impactful part of participating in a CEL course? What was the least impactful part of participating in a CEL course?
8. What else haven’t I asked you that I should have?

## References

[bibr1-10538259241309640] Brabazon H. Esmail J. Locklin R. Stirling A. (2019). Beyond employability: Defamiliarizing work-integrated learning with community-engaged learning. Engaged Scholar Journal: Community-Engaged Research, Teaching, and Learning, 5(2), 21–41. 10.15402/esj.v5i3.70364

[bibr2-10538259241309640] Braun V. Clarke V. (2006). Teaching thematic analysis. The Psychologist, 26(2), 120–123. https://uwe-repository.worktribe.com/output/937596

[bibr3-10538259241309640] Cefalu C. Kney A. D. (2020). Aspirations: Overcoming barriers to success and developing character through pre- and post-secondary school partnerships. American Society for Engineering. 10.18260/1-2-34169

[bibr4-10538259241309640] Chetty N. D. Handayani L. Binti Sahabudin N. A. Ali Z. Hamzah N. Kasim S. (2019). Learning styles and teaching styles determine students’ academic performances. International Journal of Evaluation and Research in Education (IJERE), 8(4), 610–615. 10.11591/ijere.v8i4.20345

[bibr5-10538259241309640] Choi M. M. Kuo S. W. (2009). *Social metacognition in groups: Benefits, difficulties, learning and teaching* . In Larson C. B. (Ed.), Metacognition: New research developments (pp. 117–136). Nova Science Publishers. https://scholar.google.com/scholar_lookup

[bibr6-10538259241309640] Chu M.-W. Craig H. L. Yeworiew L. B. Xu Y. (2020). Teachers’ unpreparedness to accommodate student needs. Canadian Journal of School Psychology, 35(3), 210–224. 10.1177/0829573520916610

[bibr7-10538259241309640] Cooper L. A. Kotys-Schwartz D. A. (2013). Designing the design experience—Identifying factors of student motivation in project-based learning and project-based service-learning. ASEE Annual Conference and Exposition, Conference Proceedings, *0*, 1–13. 10.18260/1-2-19396

[bibr8-10538259241309640] Corden A. Sainsbury R. (2006). Using verbatim quotations in reporting qualitative social research: Researchers’ views. *Social Policy Research Unit*. https://www.york.ac.uk/inst/spru/pubs/pdf/verbquotresearch.pdf

[bibr9-10538259241309640] Cutler H. Dorow S. (2012). *Student and community partner expectations for effective community-engaged learning partnerships*. https://files.eric.ed.gov/fulltext/EJ1001364.pdf

[bibr10-10538259241309640] Dong Y. Peng C. Y. (2013). Principled missing data methods for researchers. SpringerPlus, 2(1), 222. 10.1186/2193-1801-2-222 23853744 PMC3701793

[bibr11-10538259241309640] Dunn T. J. Baguley T. Brunsden V. (2013). From alpha to omega: A practical solution to the pervasive problem of internal consistency estimation. The British Journal of Psychology, 105(3), 399–412. https://doi.org/10.1111/https://doi.org/bjop.12046 10.1111/bjop.12046 24844115

[bibr12-10538259241309640] Flaisher-Grinberg S. (2023). Community-engaged pedagogy in the psychology classroom: Shelter dogs go to college. Teaching of Psychology, 10. https://doi.org/10.1177/00986283231191748

[bibr13-10538259241309640] Fredricks J. A. Blumenfeld P. C. Paris A. H. (2004). School engagement: Potential of the concept, state of the evidence. Review of Educational Research, 74(1), 59–109. 10.3102/00346543074001059

[bibr14-10538259241309640] Guba E. G. Lincoln Y. S. (1989). Fourth generation evaluation. Sage Publications.

[bibr15-10538259241309640] Hendrick L. Opdenakker M.-C. Van der Vaart W. (2023). Students’ academic engagement during COVID-19 times: A mixed-methods study into relatedness and loneliness during the pandemic. Frontiers in Psychology, 14(2023), 1221003–1221003. 10.3389/fpsyg.2023.1221003 37744611 PMC10514504

[bibr16-10538259241309640] Hodgson-Bautista J. Hopson R. L. Pearson J. (2022). “*A perfect storm of stress”—People for education*. https://peopleforeducation.ca/wp-content/uploads/2022/05/People-for-Education_A-Perfect-Storm-of-Stress_May-2022.pdf

[bibr17-10538259241309640] Hou S.-I. (2014). Integrating problem-based learning with community-engaged learning in teaching program development and implementation. Universal Journal of Educational Research, 2(1), 1–9. 10.13189/ujer.2014.020101

[bibr18-10538259241309640] Kolb D. A. (1984). Experiential learning: Experience as the source of learning and development, Vol. 1. Prentice-Hall.

[bibr19-10538259241309640] Kostell S. Hamshaw K. DeSisto T. Kolodinsky J. (2021). Fusing community-engaged learning & transdisciplinary curriculum for undergraduate community development education. International Journal of Community Well-Being, 4(2), 207–225. 10.1007/s42413-021-00137-3 34723122 PMC8268620

[bibr20-10538259241309640] McDonald R. P. (1999). Test theory: A unified treatment (1st ed.). Psychology Press. 10.4324/9781410601087

[bibr21-10538259241309640] Mcleod S. (2024). Kolb’s learning styles & experiential learning cycle. Simply Psychology. https://www.simplypsychology.org/learning-kolb.html

[bibr22-10538259241309640] Meyers S. A. (2009). Service learning as an opportunity for personal and social transformation. International Journal of Teaching and Learning in Higher Education, 21(3), 373–381.

[bibr23-10538259241309640] Patton M. Q. (2015). Qualitative research & evaluation methods. Sage Publications, pp. 4.

[bibr24-10538259241309640] Pfingstgraef K. Yates J. Rose H. Mantler T. (2024). The impacts of undergraduate student developed resources on elementary students and teachers: A scoping review. *Western Undergraduate Research Journal*. Submitted.

[bibr25-10538259241309640] Pomerantz N. (2006). Student engagement: A new paradigm for student affairs. College Student Affairs Journal, 25(2), 176–185.

[bibr26-10538259241309640] Quirkos (2021). *Qualitative analysis software*. Version 2.4.2 [software]. https://www.quirkos.com

[bibr27-10538259241309640] R Version 4.2.2 (2022). *The R Foundation for Statistical Computing Platform* .

[bibr28-10538259241309640] Sansone G. Fallon B. Birken C. Miller S. P. Gallagher-Mackay K. Sajedinejad S. Jenkins J. Davies S. (2021). Effects of school closures during the COVID-19 pandemic on achievement gaps and learning inequalities: Policy implications. Policy Bench, Fraser Mustard Institute of Human Development, University of Toronto. https://socialwork.utoronto.ca/wp-content/uploads/2021/07/Policy-Bench-Brief-School-Closures-and-Achievement-Implications-Final-July9.pdf

[bibr29-10538259241309640] Seaman J. Brown M. Quay J. (2019). The evolution of experiential learning theory: Tracing lines of research in the JEE. Journal of Experiential Education, 40(4), NP1–NP21. https://doi.org/10.1177/1053825916689268

[bibr30-10538259241309640] Shinneman A. L. C. Loeffler S. Myrbo A. E. (2020). Self-guided field trips allow flexibility in undergraduate student introductory field experiences. Journal of Geoscience Education, 68(4), 371–379. 10.1080/10899995.2020.1768006

[bibr31-10538259241309640] Stanton J. D. Sebesta A. J. Dunlosky J. (2021). Fostering metacognition to support student learning and performance. CBE life Sciences Education, 20(2), fe3. 10.1187/cbe.20-12-0289 PMC8734377 33797282

[bibr32-10538259241309640] Surapaneni K. M. (2023). “Metapad” (metabolic pathways decoded)—A gaming innovation to ease the complexity of metabolic pathways by promoting self-directed, active, participatory learning in small groups. BMC Medical Education, 23(1), 1–23. 10.1186/s12909-023-04587-5 37626407 PMC10464076

[bibr33-10538259241309640] UNESC (2023). UNESCO’s education response to COVID-19. https://www.unesco.org/en/covid-19/education-response/initiatives

[bibr34-10538259241309640] Walker J. P. Rocconi L. M. (2021). Experiential learning student surveys: Indirect measures of student growth. Research & Practice in Assessment, 16(1), 21–35.

[bibr35-10538259241309640] Yates J. Davidson C. A. Orchard T. Burke S. M. Mantler T. (2024). “I watched my child become half of the person they were”: Emergency remote learning experiences among primary caregivers of elementary school children during the COVID-19 pandemic. *Journal of Child and Family Studies*. Submitted. 10.1177/00220574251349795 PMC13046253 41939731

[bibr36-10538259241309640] Zoom (2024). Version 5.17.10. *Zoom Video Communications* [software]. https://zoom.us/

